# The structural evolution of undisturbed loess due to water infiltration

**DOI:** 10.1038/s41598-024-65838-z

**Published:** 2024-06-27

**Authors:** Jianqi Zhuang, Jiaxu Kong, Yi Zhu, Jianbing Peng

**Affiliations:** 1 College of Geological Engineering, Geomatics of Chang’an University, Xi’an, China; 2 College of Land Engineering, Chang’an University, Xi’an, China

**Keywords:** Loess, Infiltration, Internal erosion, Structural evolution, Particles and pores, Geomorphology, Hydrogeology, Mineralogy, Sedimentology, Civil engineering

## Abstract

Loess structure is the physical key factor that determines its stability and consists of macro-pores, loose texture, and water sensitivity. The structural change characteristics and effects of the undisturbed loess before and after water infiltration are studied using mechanical CT and simulation tests in order to study the structural change process within the undisturbed loess caused by water infiltration. The change in particle state is as follows: the peak frequency point of the equivalent diameter of the loess particles after infiltration ranged from 16.75 to 23.76 μm, and the eroded fine particles consisted primarily of fine particles. The smaller loess particles are removed by water infiltration resulting in coarsening of soil particles. The sphericity of the loess particles gradually changes from spherical pores to angular and dendritic pores. The particle inclination angle transitions to a range greater than 70°, and its proportion is approximately 61%. The change in pore structure is as follows: The loess porosity after infiltration increased by approximately 20%, and the increase in the pore area ratio of the mesopores and the macropores was higher than that of the micropores. Additionally, the small pores increased by more than 5 times the original state of the undisturbed loess. The connected pores expanded less than 60% of the initial state to more than 90% after infiltration, thus, increasing the dominant seepage channel of the undisturbed loess. These changes in particle and porosity further increase the water filtration intensity and promote the migration of fine particles (mainly silt particles), linking loess catastrophes and are the leading cause of loess settlement and slope instability. The process of water infiltration into the loess, the mechanism of loess collapsibility, and the influence of salinity on the loess structure and strength are discussed in this study.

## Introduction

Loess is a silt-dominated, calcium-rich, brown-yellow deposit formed by aeolian processes since the Quaternary period^1^. Loess is widely distributed worldwide, especially in China. The Chinese Loess Plateau accounts for approximately 6.6% of China's land area. The loess area is the origin of Agricultural Culture and is North China's main crop-growing region^1^. Due to its structural characteristics, the loess has typical characteristics of large pores, the development of vertical joints, easy disintegration, and collapsibility. Thick loess deposits are considered "Kast soils" due to their susceptibility to internal erosion, including transport erosion and dissolution^2–5^. These typical characteristics make the loess highly sensitive to environmental water changes, resulting in mass movement. More than 85% of the loess area's geological hazards and engineering failures are caused by water. The geohazards cause a large amount of soil loss, infrastructure damage, and the destruction of good farmland, resulting in losing large-scale land area in loess tableland^6^. Rainfall and irrigation are the main factors for the frequent occurrence of geological hazards within the loess area^2,4^. During the process of inducing geological hazards due to water, the first issue is water infiltration, meaning how water enters the loess and what kind of changes happen when it enters the loess, which is the cause of loess catastrophes.

The direct consequences of water entering the loess are collapsibility and settlement due to structural collapse, which is a unique engineering geological property of loess and is related to numerous geological hazards and engineering failures within the loess area^7–9^. Therefore, the structural changes after water enters the loess are essential in studying loess geohazards. Internal erosion includes transport erosion and chemical dissolution that occurs when the water infiltrates the loess^9^. The fine particles (clay and silt) and chemicals move within the matrix of the macro-pores under seepage flow resulting in loess skeletal destruction^9–11^. The loess structure is the key factor in maintaining the high strength of the loess while the loess structure is damaged, the physical and mechanical properties of loess, such as strength, deformation, and permeability, will slowly change over time; therefore, deteriorating loess engineering properties represents a risk to the safety of the loess slope and land subsidence via changes in the loess structure^9,12^.

The microstructure of loess is formed during the process of diagenesis after the loess is deposited^13,14^. The unique engineering geological properties of loess collapsibility are closely related to the microstructure of the loess. Loess collapsibility and failure result from microstructure damage^9,12,14^. The soil's microstructure determines the loess's physical, chemical, and mechanical properties, affecting the soil's macroscopic stability. The instability of a soil's microstructure is closely related to its stress state, water content, geometric size, and wet and dry cycles. Vibration, stretching, compaction, and loading of the loess can lead to microstructural damage^9,14–16^. At the beginning of the twentieth century, Terzaghi studied soil microstructure and found that soil has a typical nest-like structure^17,18^. Since the 1950s, scholars have analyzed the microstructure of loess from particle morphology, contact relationship, pore morphology, and cementing materials, thus, significantly expanding the knowledge within this field of research^19–22^. In recent years, with the advancement of science and technology, such as the rapid developments in electron microscopy technology, the study of soil microstructure has grown significantly and therefore has attracted the attention of researchers on a global scale^23–28^. How to quantitatively describe the structure of the soil and realize the correlation between the soil's macroscopic and mechanical characteristics has also gained much attention^14,23,25,26,28^. Currently, the common observation methods of soil microstructure include optical and scanning electron microscopes (SEM), mercury intrusion (MIP), and computed tomography (CT). However, the images obtained by optical microscopy and SEM are all two-dimensional images, and the results depend on the chosen observation direction^29–31^. The MIP technique may cause damage to the sample, and the obtained pore size results do not reflect the true size distribution^25^. CT imaging can obtain the true geometric features of the particles and pores and can perform three-dimensional reconstruction and visualization through non-destructive, quantitative, reproducible, and high-resolution analysis^14,25,32^. In recent years, many scholars have used CT scanning to construct the three-dimensional structure of the soil, as well as analyze the change characteristics of rock and soil microstructures under the influence of hydraulic and other factors^14,25,32^. The study of soil microstructure gradually changed from macro to micro and from qualitative to quantitative. For example, Li et al^32^ carried out CT scans of loess and found that the large structural units of loess show an apparent concentration in the vertical direction. Analyzing the loess pore structure's geometric parameters shows that loess is a highly anisotropic geological material. These research results established the correlation between the soil microstructure parameters and the macroscopic mechanical characteristics. Although the above studies have carried out very detailed studies on the structural changes caused by the change of porosity, the change of porosity is important, and the change of particles still plays a key role in this process, and the change of particles will affect the strength and structure of the soil, which has been neglected in the past^7,33–35^. The subsequent development will be to explain the catastrophic mechanism of loess through the process of structural change and three-dimensional information^36^. Meantime, there is a lack of research on the structural change process of soil, especially the soil's structural change during water infiltration.

The type of water infiltration process that enters the loess, what happens when water enters the loess, the physical changes in its particles and pores, as well as the impact of these changes on loess geohazards have always been highly controversial^4,6,36^. The structure of loess is the key factor that gives loess its unique macro-pore characteristics, loose texture, and water sensitivity^14,23,25,32,37^. Furthermore, water is the primary reason for various geohazards within the loess area. Studies surrounding the structural change characteristics of the loess before and after water infiltration can provide technical support for loess geohazard mitigation. In this paper, the process of water infiltration into the loess is carried out by designing undisturbed loess infiltration tests. The structural change characteristics of the undisturbed loess before and after water infiltration are studied using CT imaging. The water infiltration process effect is studied using numerical simulation and mechanical tests. The influence of the soluble salt content of the loess on structure and loess failure is discussed. The results provide a reference in demonstrating the water's failure mechanism process.

## Sampling and methods

### Sampling

**Figure 1:**
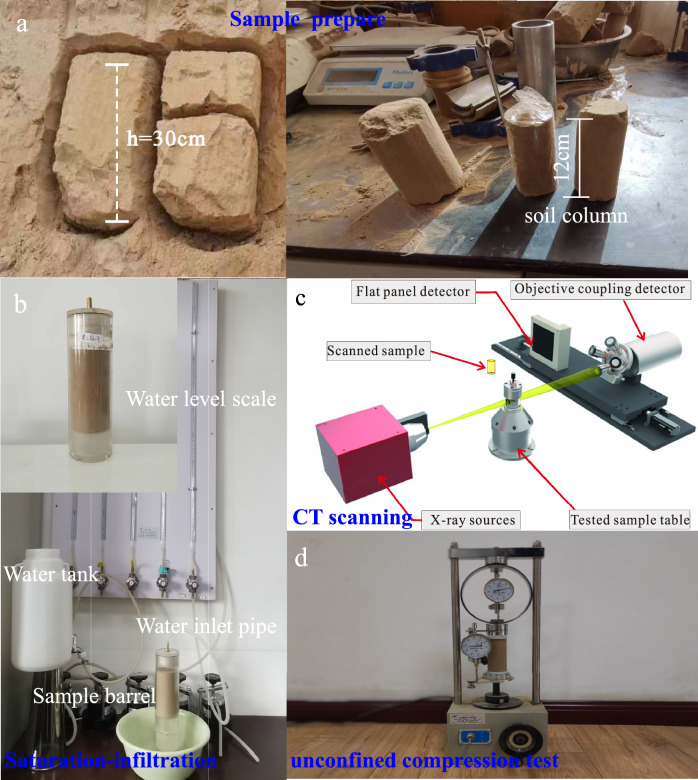
The test flow chart (a: loess sampling; b: infiltration test; c: CT test; d: unconfined compression tests).

Twenty undisturbed loess block samples are obtained by manual excavation from the Qingyang and the Dongzhi Tableland, located in the Middle East of the Chinese Loess Plateau, a typical loess region. These soil samples were obtained from a depth of 2 m from the tableland surface (the land use is grass) to obtain the undisturbed loess soil. Field monitoring shows almost no change in soil water content with a rainfall depth greater than 2 m. Each soil sample was cut into approximately 20 cm-sized soil blocks. These twenty soil samples were obtained from the exact location, ensuring the consistency of the test samples. These twenty samples were then used to conduct separate experiments. The loess soil samples belong to Manlan loess. The blocks were wrapped tightly with black plastic bags and tape sealed to protect the blocks. The samples were marked and transported to the laboratory for analysis (Fig. 1a). The other loess samples located in Heifangtai tableland, West of the Chinese Loess Plateau, were obtained following the same method in order to be able to analytically compare the influence of material composition on the physical properties of loess. The undisturbed loess's basic physical properties, mineral composition, strength, and microstructure characteristics were obtained using indoor tests following the geotechnical test standard methods.

The soil particle size distribution was measured using a Bettersize 2000 laser, and the basic physical indexes of the soil samples are measured according to geotechnical test standards and shown in Table 1.

**Table 1:** Physical characteristics of the loess sample.

Loess sample	Gravimetric water content	Dry density	Plastic limit	Liquid limit	Particle size (%)		
	*w*/(%)	*ρ*/(g·cm^-3^)	*W*_*P*_ /(%)	*W*_*L*_/(%)	> 0.075 mm	0.005–0.075 mm	< 0.005 mm
Qingyang	10.1	1.52	17.2	30.7	8.58	72.67	18.75
Heifangtai	8.9	1.46	18.3	27.5	17.22	74.22	8.56

### Testing methods

For the undisturbed loess sample, we cut the undisturbed soil into a soil column at a diameter of 61.8 mm and a height of 120 mm and then brushed a thin layer of transparent waterproof glue onto the side wall. The distilled water in the bucket is supplied to the soil column through a rubber tube. The water head height above the soil column is maintained at 1–2 mm, simulating the precipitation process and preventing differences in water high heads until the wet peak reaches the bottom of the soil column. The amount of water seeping from the soil column daily was measured and recorded. The settlement deformation in the vertical direction of the soil column was observed and recorded. After 3 days of infiltration, the test was terminated, simulating the water infiltration process into the loess.

After the infiltration tests, the vapor that sublimated from a solid to a gaseous state in the soil sample was extracted using a freezing method to dry the soil sample. Next, the infiltrated soil sample was removed from the PVC pipes (Fig. 1b). The fine particles that seeped from the sample were collected and dried to test the mineral composition. The X-ray diffraction patterns of the samples were acquired using an Empyrean X-ray diffractometer. The X-ray diffraction patterns of the soil particles were analyzed using Jade software to obtain their mineral composition and content^38^. The seepage water was filtered using a 0.45 μm filter paper and analyzed using ICP-AES mass spectrometry and ion chromatography to determine the concentrations of the main cations and anions within the samples^9,38^. The Agilent 725 ICP-AES inductively coupled to a plasma emission spectrometer was used to measure the concentration of Na^+^, K^+^, Ca^2+^, and Mg^2+^ within the soil samples; the concentration of ${\text{Cl}}^{ - } ,{\text{NO}}_{{3}}^{ - } ,{\text{SO}}_{{4}}^{2-}$ in the soil samples were tested using an ICS1000 ion chromatograph. A titration method was used to test the concentration of ${\text{CO}}_{3}^{2 - } ,{\text{HCO}}_{3}^{ - }$ within the loess samples.

The undisturbed loess specimens were scanned using nanoVoxel-5000 CT equipment to investigate the influence of water infiltration on the microstructural change of the loess (Tianjin Sanying Company)^14^. The equipment can not only observe the three-dimensional appearance and internal structure of the undisturbed loess while ensuring the integrity of the samples but can also realize the three-dimensional visualization of the observation results. The CT equipment comprises a high-resolution X-ray source, a detector, and a scanning system. The working voltage and current are set to 150 kV and 500A, respectively, and the scanning mode adopts the circular track mode. The pixel size of the flat-panel detector is 49.5 μm, the scanning resolution is 0.2 μm, and each scanned image is composed of 5888 × 4608 pixels (Fig. 1c). In this study, the resolution of the undisturbed loess sample is 10 microns, and the gray value of the two-dimensional slice is 0–255 microns. The binary image is obtained by clipping, median filtering, noise reduction, and threshold segmentation. The white regions are particles, and the black regions are pores. The 3D structural maps of the undisturbed soil samples, and the undisturbed soil samples after infiltration were obtained.

In order to assess the influence of infiltration on the strength of loess, unconfined compression (UC) tests were conducted following the standard geotechnical test methods^39^. The UC tests were carried out utilizing a YYW-2 strain control type unconfined pressure gauge, controlling the gauge plate's rate at a constant speed of 2.4 mm/min. During the tests, the stress of the samples was monitored, and the measurement process concluded upon complete specimen failure (Fig. 1d).

### Analysis method

The CT images were binarized, and the segmentation threshold selection significantly influenced the characteristic analysis of the particles and pores^14,25,40^. The porosity or the pore area ratio is the most accurate way to determine whether the threshold is reasonable. The CT image is a 16-bit grayscale image with 65,536 color values. Comparing the segmented sample pores with the undisturbed loess pores, 11,280 was obtained as the best segmentation threshold point^41^. Using Avizo software, the micron-scale CT 3D structure data of the undisturbed loess, including diameter, area, angle, and sphericity of the pores and particles, were calculated using median filtering, noise reduction, and threshold segmentation.

Equivalent diameter is the average length of the diameters measured at 2-degree intervals and was passed through the center of the object and calculated as^26^:

1 $${D}_{pore}=\frac{\sum_{i=1}^{J}{d}_{i}}{J}$$

where, *d*_*i*_ is the equivalent diameter, and *J* is the number of particles and pores. When the roughness of the object or its pore size is reduced, its specific surface area (SSA) also increases^41^. The specific surface area is the surface area per unit volume and can be calculated as:

2 $$SSA=\frac{{A}_{3Dpore}}{{V}_{3Dpore}}$$

where, *A*_*3Dpore*_ is the pore area, and *V*_*3Dpore*_ is the pore volume. The sphericity reflects the morphological characteristics of the pore structure or the loess particles. Significant differences exist in the sphericity index of different sizes. The larger the sphericity, the closer the shape is to a sphere. The expression is shown in Eq. (3).

3 $$SPH=\sqrt{\frac{4{V}_{3D}}{\pi {L}_{3D}}}$$

where, *SPH* is sphericity, and *L*_*3D*_ is the pore long-axis size. The sphericity value ranges from 0 to 1. The inclination angle represents the angle between the pore or particle's long axis and the horizontal plane's positive direction. The directional frequency (*P*_*i*_(*α*)) can be used to quantitatively analyze the distribution characteristics of the pore or particle dip; its expression is shown in Eq. (4) ^14^.

4 $${P}_{i}\left(\alpha \right)=\frac{{m}_{i}}{{M}_{i}}\times 100\%$$

where *m*_*i*_ is the number of pores or particles within a specific range, *M*_*i*_ is the total number of pores or particles, and *α* is the range of the inclination angles^14^. The pore area ratio (PAR) represents the ratio of the pore area to the total area of the binary image and is calculated as Eq. (5) ^16^:

5 $$PAR=\frac{{A}_{total pore}}{{A}_{total}}\times 100\%$$

where, *A*_*total pore*_ is the pore area, and *A*_*total*_ is the total area of all binary images.

### Simulation method

This study's simulation of absolute permeability was conducted using the Avizo software platform with the XLab module. Specifically, the pores in the loess sample were considered when simulating the absolute permeability, as mentioned by Yu et al^40^. The fluid flow occurring in the pores of the loess sample was simulated using the Navier–Stokes equation. In this simulation, pure water was used as the flowing medium, with parameters including a density of ρ = 1000 kg/cm^3^ and a viscosity of μ = 0.001 pa.s. The lower inlet pressure was set at 1.3*10^5^ Pa, while the upper end functioned as the outlet. The volumetric flow rate of water passing through the tailings model was obtained by volumetrically dividing the seepage velocity at the outlet boundary.

Absolute permeability can be defined as the measure of a porous material's ability to transmit a single-phase fluid. Following Darcy's law, it represents a constant coefficient that establishes the relationship between fluid flow and the material's parameters (Eq. 6).

6 $$\frac{Q}{S}=-\frac{k}{\mu }\frac{\Delta P}{L}$$

where *Q* is the global flow rate that goes through the porous medium (m^3^/s), *S* is the cross-section of the sample which the fluid goes through (m^2^), *k* is the absolute permeability (m^2^), *µ* is the dynamic viscosity of the flowing fluid (Pa·s), Δ*P* is the pressure difference applied around the sample (Pa), *L* is the length of the sample in the flow direction (m).

## Results

### Particle Characteristics

**Figure 2:**
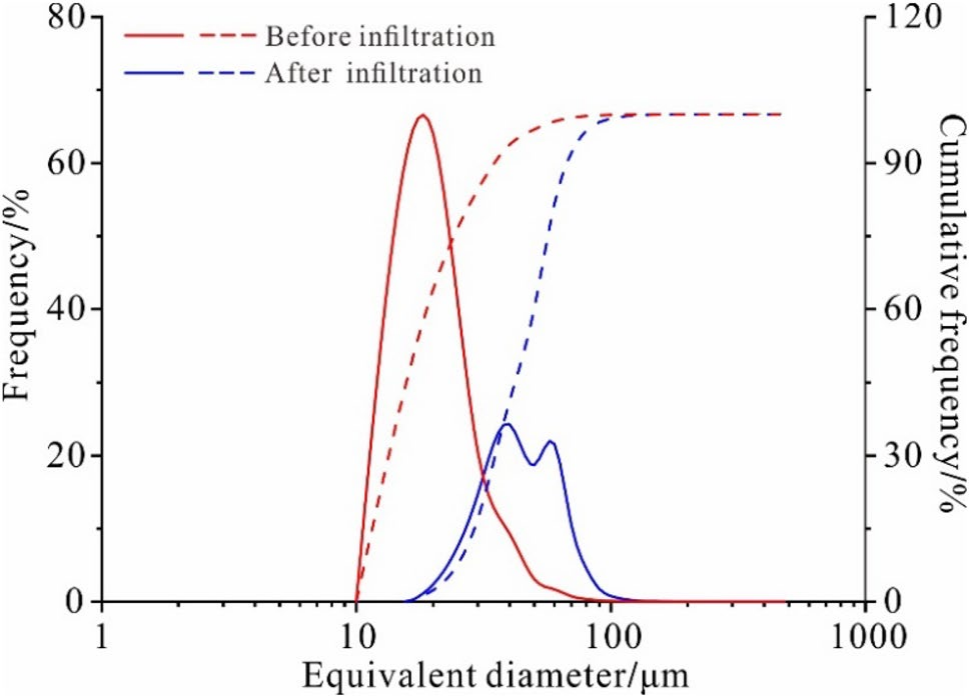
The equivalent diameter distribution curve of particles before and after water infiltration (solid line is frequency and dashed line is cumulative frequency).

The equivalent diameter distribution curve of the undisturbed loess shows single-peak distribution characteristics with the natural and saturated states. The loess particles smaller than 40 μm account for 60% of the total particles. The equivalent diameter distribution curve has the characteristics of dispersive distribution after infiltration of the undisturbed loess. The loess particles larger than 30 μm account for approximately 70% due to a large number of fine particles lost during the infiltration (Fig. 2). The test results show that the peak frequency point of the equivalent diameter of the loess particles after infiltration range from 16.75 to 23.76 μm. The distribution frequency curve of the equivalent diameter of the particles moves to the right side of the coordinate axis, indicating that the equivalent diameter of the undisturbed loess increases significantly after infiltration. The infiltration water removes the small loess particles, resulting in a relative increase in large particle content.

**Figure 3:**
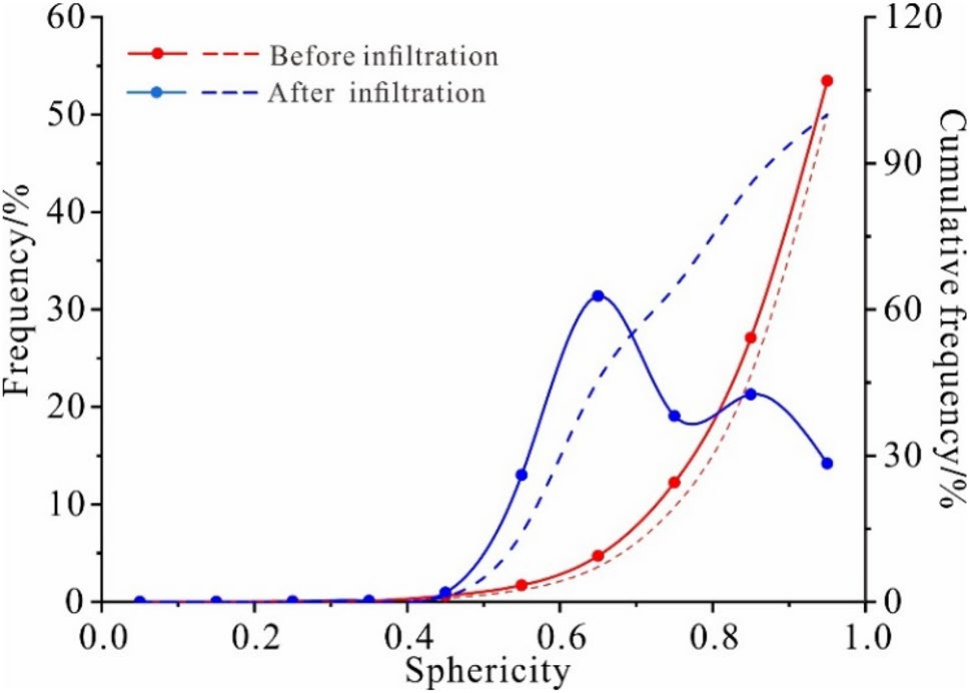
The sphericity index distribution curve in natural and permeable test states (solid line is frequency and dashed line is cumulative frequency).

The sphericity index can be divided into spherical (1–0.8), angular (0.8–0.6), and dendritic pores (0.6–0). Figure 3 shows the undisturbed particle sphericity distribution curves in natural and permeable test states. The particle sphericity distribution curves show that the undisturbed loess's particle sphericity changes after infiltration, and the sphericity index of the loess particles after infiltration differs from the undisturbed loess. The sphericity index ranges from 53.45 to 31.37%, and the sphericity of the loess particles gradually changes from spherical pores to angular and dendritic pores. Within the loess particle composition, the fine particles' equivalent diameter is usually small, and the sphericity is relatively large, consisting of spherical or quasi-spherical pores. This indicates that during the water infiltration process, the loss of fine particles of the undisturbed loess is large, resulting in the primary composition of the loess being coarse particles and other substances in the undisturbed loess after infiltration.

**Figure 4:**
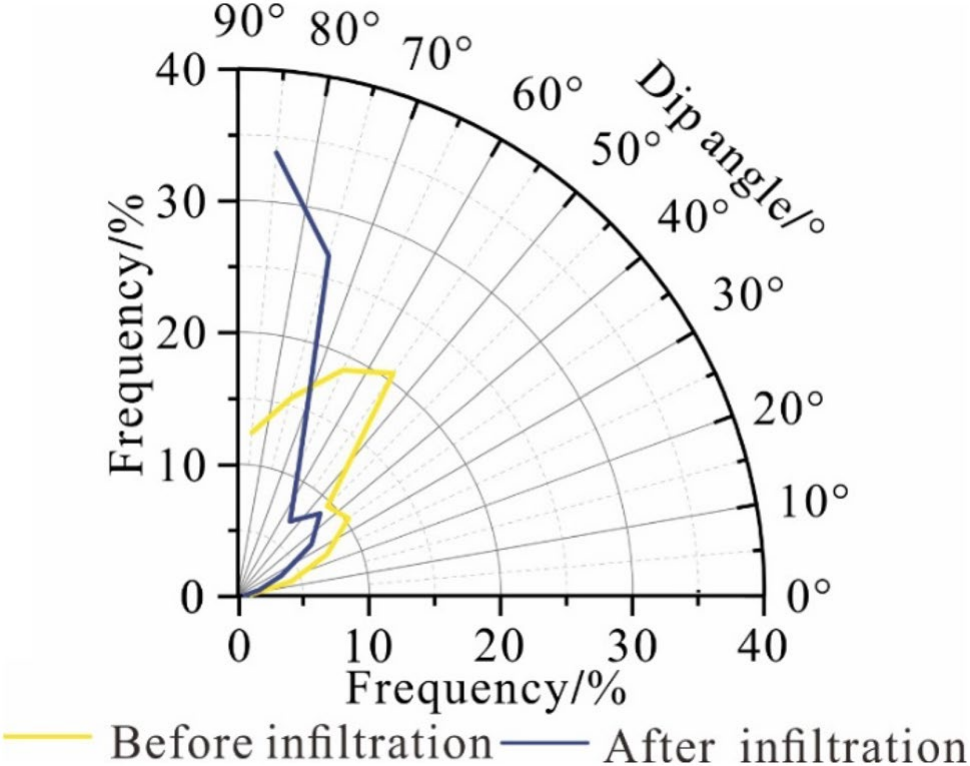
The particle inclination angle distribution before and after infiltration.

The particle inclination angle is primarily distributed in the range of 50° to 60°, and after the infiltration test, the hydrodynamic driving effect leads to the rearrangement of the particles in the undisturbed loess sample. The particle inclination angle transitions to a range greater than 70°, and its proportion is approximately 61% (Fig. 4). The infiltration process shows that the infiltration causes the particle arrangement of the undisturbed loess to change. Furthermore, the directional arrangement along the water infiltration direction becomes increasingly apparent.

The particle changes in the undisturbed loess during the process of infiltration reflect that the process of water entering the undisturbed loess is a process that changes the particles' state. The water infiltration process significantly promotes the change of the loess structure as well as the fine particles that were carried away through internal erosion. During this process, the equivalent diameter of the particles becomes larger, and the particle's long axis gradually arranges along the water infiltration direction. These changes in particle morphology further increase the intensity of water infiltration and promote the migration of fine particles.

### Characteristics of pores

**Figure 5:**
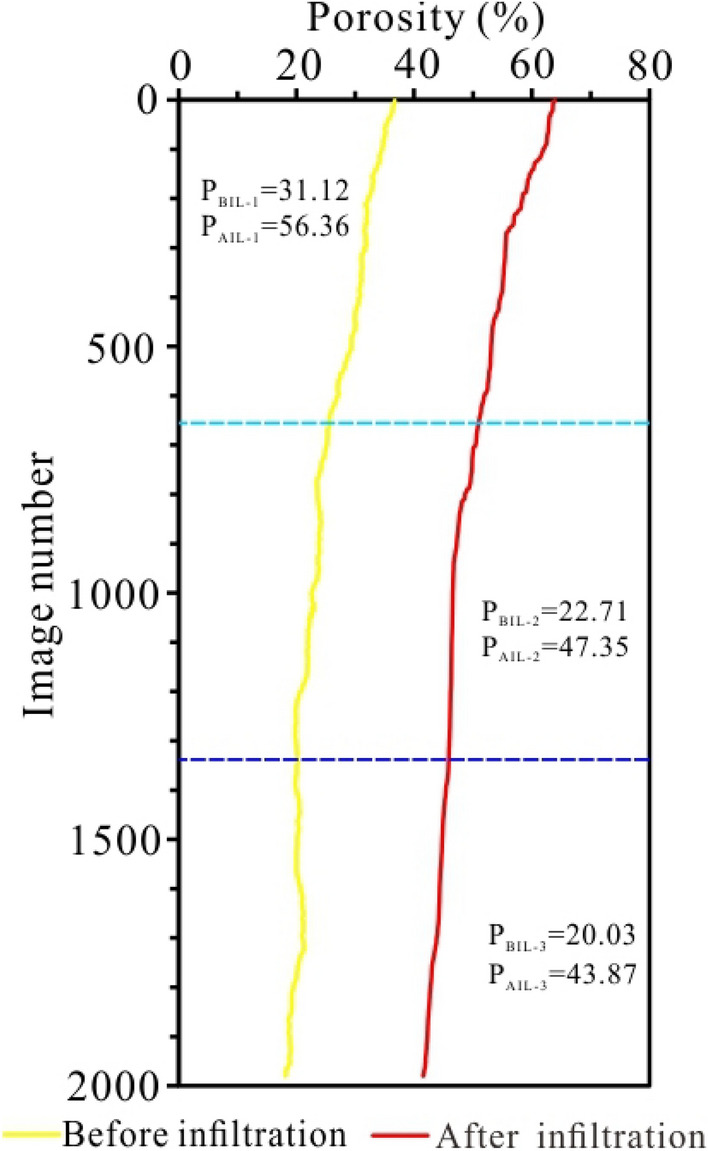
The plane porosity distribution from sample top to bottom before and after infiltration.

The plane porosity of the undisturbed loess increases after the permeability test. The undisturbed loess had a looser structure and primary pores, leading to a continuous increase in plane porosity. During infiltration, the pores of the loess samples continued to increase; however, with the downward migration of the fine particles, the pores of the loess sample show the characteristics of large upper parts and small lower parts (Fig. 5). The porosity of loess after infiltration increased by approximately 20% on average. Among them, the loess's upper, middle, and lower pores increased by approximately 25%, 24%, and 23%, respectively. Therefore, during infiltration, the pores of the loess samples continued to increase; however, with the fine particles' downward migration, the loess sample's pores show the characteristics of large upper parts and small lower parts (Fig. 5).

**Figure 6:**
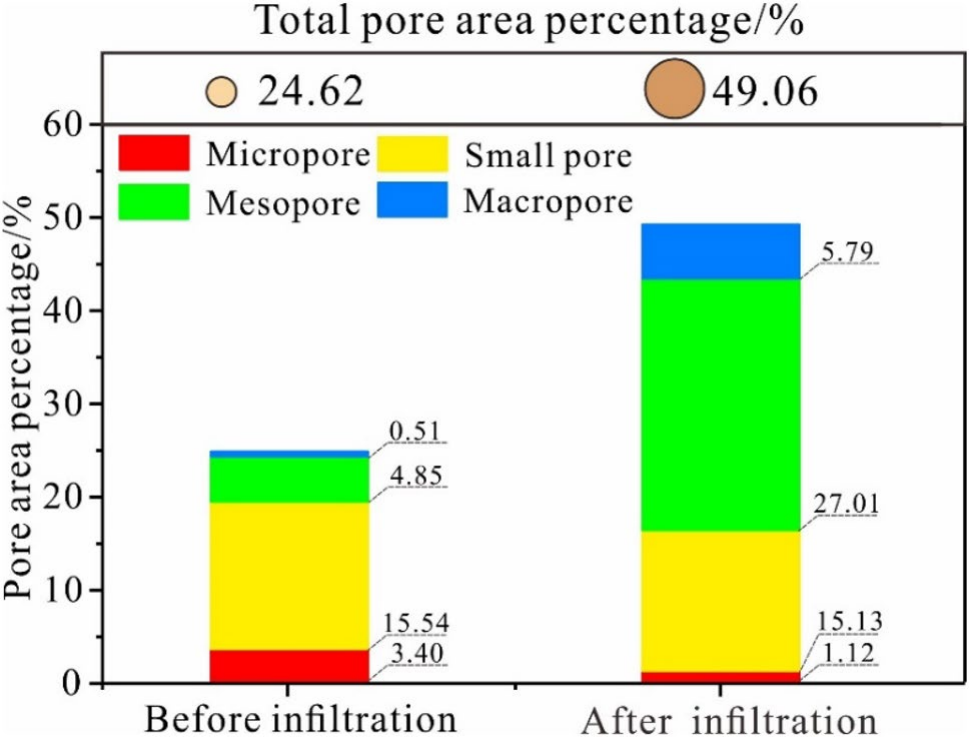
The evolution characteristics of the total pore area ratio before and after infiltration.

According to the relationship between the loess structure and the loess particles, the pores of the loess can be divided into four categories. Micropores (0-20 μm) are located between the loess particles, and their equivalent diameter is smaller than the size of the loess particles. Small pores (20–40 μm) are pores within the skeleton of the loess, and their equivalent diameter is nearly the same as the size of the loess particles^14,32,42^. Micropores and small pores are stress-bearing areas in the soil skeleton. Mesopores (40–100 μm) are distributed between the aggregate particles, and their size is twice that of the small pores. Macropores (> 100 μm) primarily include wormholes, collapsibility, porosity, and joint fissures^32,43^. Figure 6 shows that the total pore area ratio of the undisturbed loess consists of the pore area ratio of the small pores in the initial state and the total pore area ratio of the undisturbed loess, which increases to 49.06% after the infiltration tests. During this process, the increase in the pore area ratio of the mesopores and the macropores is higher than that of the micro and small pores. Among them, the increase of small, micro, and macropores are inconsistent. The increase of micro and macropores is the most apparent, with 0.51% and 4.85% of the total original state increasing to 5.79% and 27.01% after infiltration, respectively, having an increase of more than 5 times the original state of the undisturbed loess.

Water infiltration not only causes the macropores' expansion but also significantly impacts the micro and small pores. Meso and macropores are the primary reasons for the deterioration of the soil structure; therefore, the loess sample's structure develops from a dense to a loose state.

**Figure 7:**
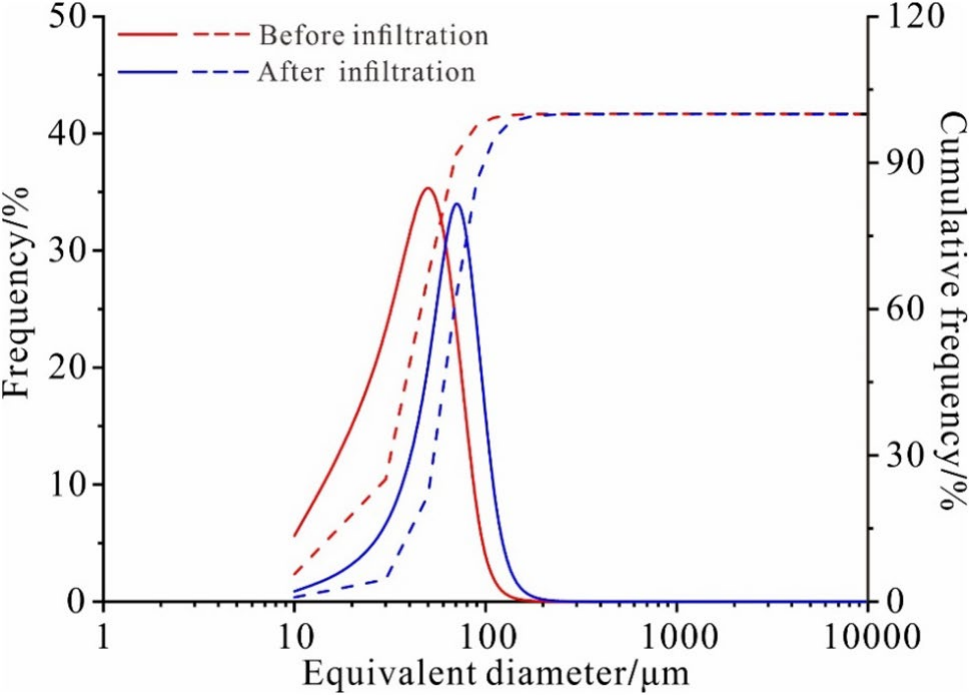
The pore equivalent diameter distribution curve before and after infiltration (solid line is frequency and dashed line is cumulative frequency).

The peak value of the pore distribution curve shifts to large pores, which range from 40 μm in the original state to 70 μm after infiltration, indicating that the soil structure is damaged. Furthermore, the particles are rearranged due to the continuous internal erosion resulting in an increase in meso and macropores within the undisturbed loess (Fig. 7). It is demonstrated that during the process of water infiltration, the increase of macropores is conducive to the formation of dominant infiltration, promoting the process of water entering the loess, increasing the internal erosion of the loess, and forming the dominant water loess seepage channel. The formation of the dominant seepage channel is beneficial to the collection of water and reduces the potential erosion of water in the non-dominant seepage channel of the loess. Therefore, the increase of macropores has a dual impact on the loess structure. On the one hand, the increase of macropores is conducive to forming dominant channels and promotes the occurrence of slip catastrophes. On the other hand, the increase of macropores is beneficial to the collection of water in order to reduce the potential erosion of water in other areas as well as reduce the occurrence of settlement.

**Figure 8:**
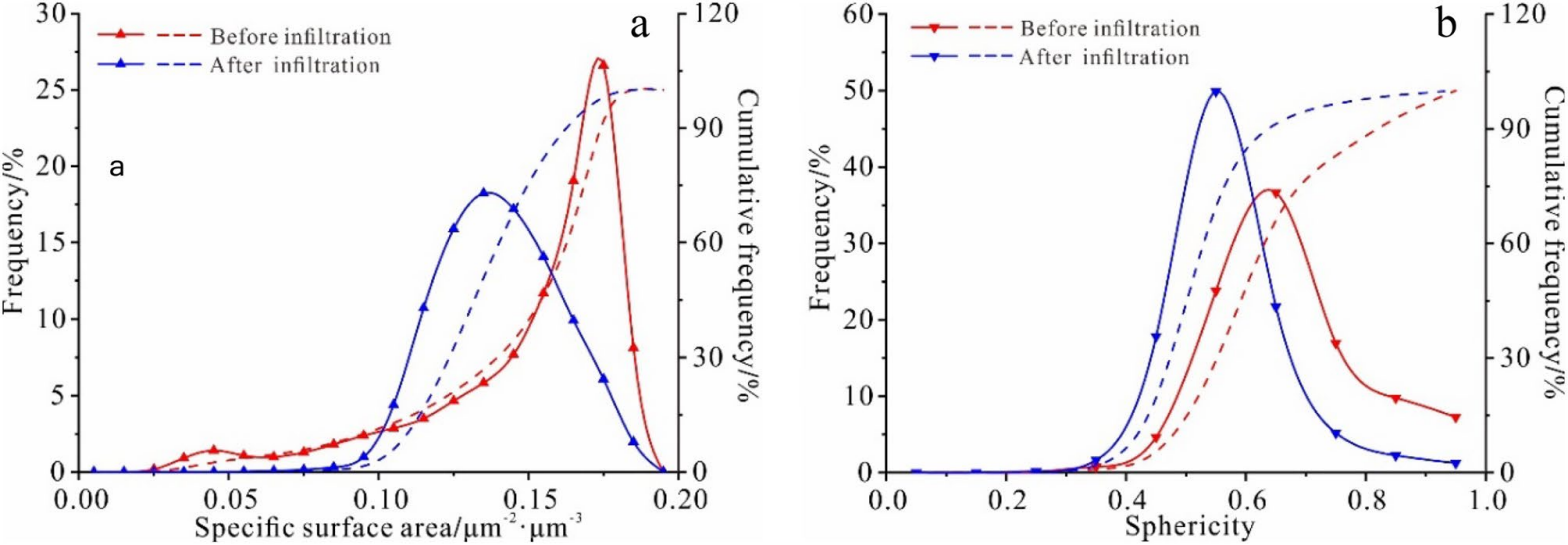
The specific surface area (**a**) and sphericity (**b**) distribution curves before and after infiltration (solid line is frequency and dashed line is cumulative frequency).

The specific surface area is a quantitative index that reflects the tightness or looseness of the soil structure. The larger the value reflects, the smaller the pore size, the relative stability, and the dense material structure^42,43^. The specific surface area of the undisturbed loess decreases after the infiltration tests. The reduction of the specific surface area of the undisturbed loess indicates that the undisturbed loess structure is easily destroyed under water infiltration (Fig. 8a).

The sphericity of the micropores and the small pores is higher than 0.8, indicating a quasi-spherical shape. Because the fine particles continuously migrate from the loess during the infiltration test resulting in a decrease of the micro and mesopores. The morphologies of the meso and the macropores are agglomerated or dendritic, having a sphericity value of less than 0.6 (Fig. 8b), which eventually leads to a continuous decrease of the sphericity value of the undisturbed loess. Furthermore, the pore morphology continues to change from spherical to aggregated or dendritic. Meanwhile, macropores are usually the dominant water infiltration channel and significantly impact the stability of the soil's structure.

**Figure 9:**
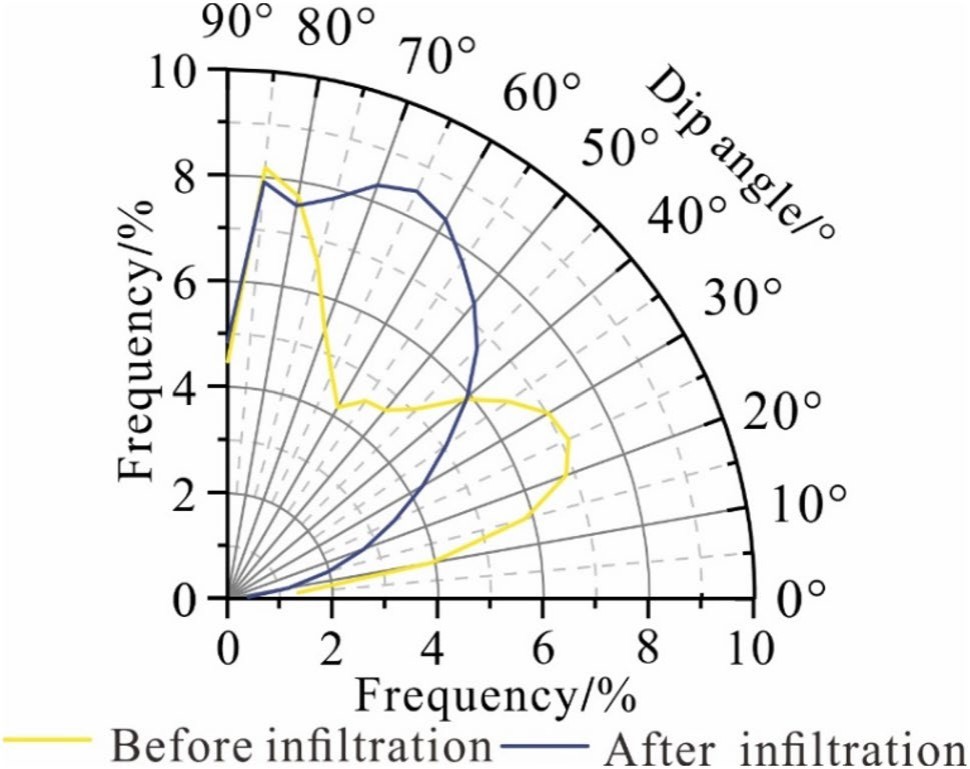
The pore inclination angle distribution before and after infiltration.

The pore inclination angle of the undisturbed loess tends to transition in the vertical direction. The frequency of pores greater than 60° increases significantly, indicating that water infiltration forms a large-scale channel conducive to water flow within the loess after the infiltration test (Fig. 9). This change of pore inclination angle of the undisturbed loess is more conducive to the formation of fixed preferential seepage channels, promoting water infiltration and forming preferential seepage.

### Pore connectivity characteristics

**Figure 10:**
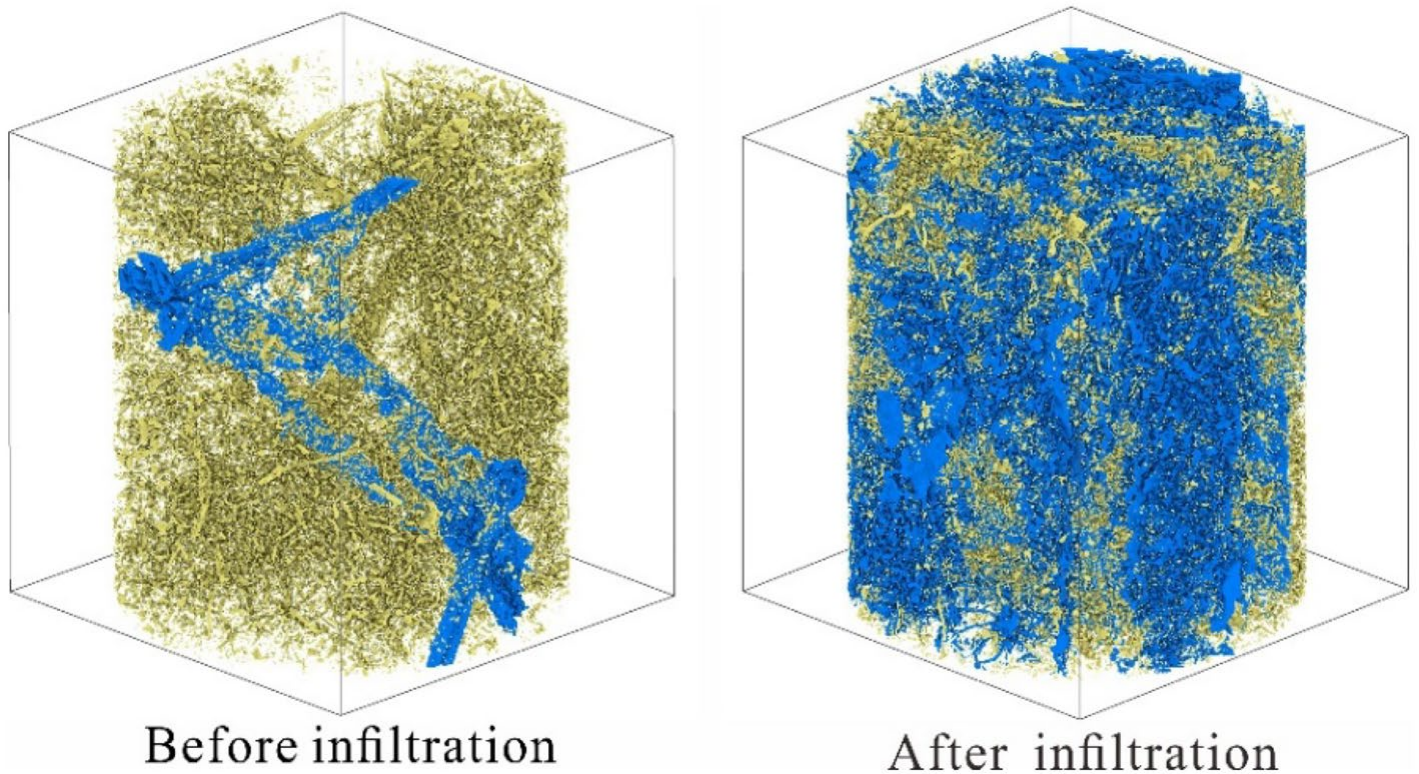
The connected and isolated pore distribution before and after infiltration.

**Figure 11:**
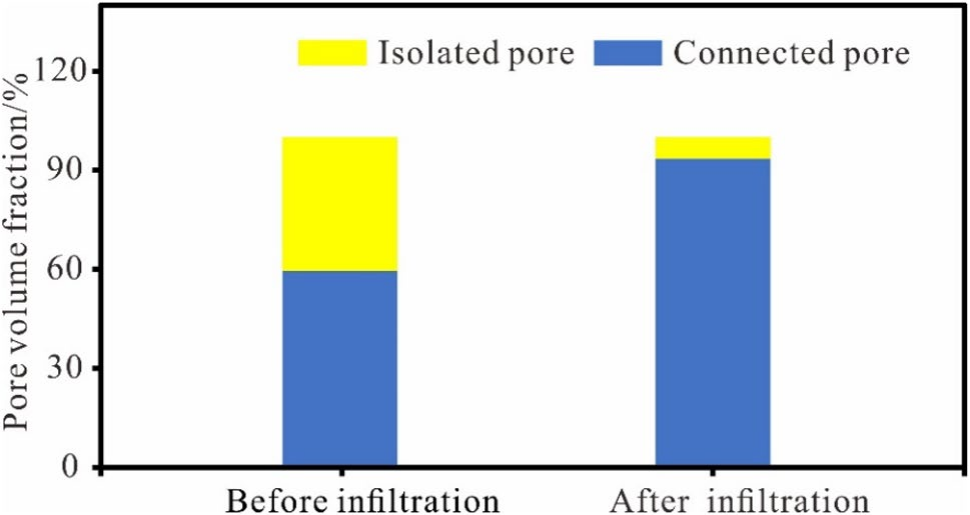
The connected and isolated pore distribution before and after infiltration.

After the infiltration tests, the undisturbed loess's connected and isolated pore distribution models were established using Avizo software. It was found that the pore structure of the undisturbed loess consists primarily of connected pores. The connected pore structures increased after the infiltration test in the undisturbed loess. In contrast, after the infiltration test, the complex connected pore structure of the undisturbed loess increased, and the isolated pores decreased (Fig. 10). The connected pores expanded from < 60% of the initial state to > 90% after infiltration. Figure 11 shows that the total porosity of the undisturbed loess increases after the infiltration tests, and the increase in total porosity of the undisturbed loess is primarily due to an increase in the connected porosity. The increase of these connected pores further increases the dominant seepage channel of the undisturbed loess, promoting the formation of dominant seepage, and is conducive to the formation of large-scale collapse and sliding geohazards along this dominant channel. This phenomenon is also common in the field. Many loess slopes are distributed with many subsurface erosion caves and connecting pores, providing a dominant channel for water seepage and promoting loess gully erosion.

**Figure 12:**
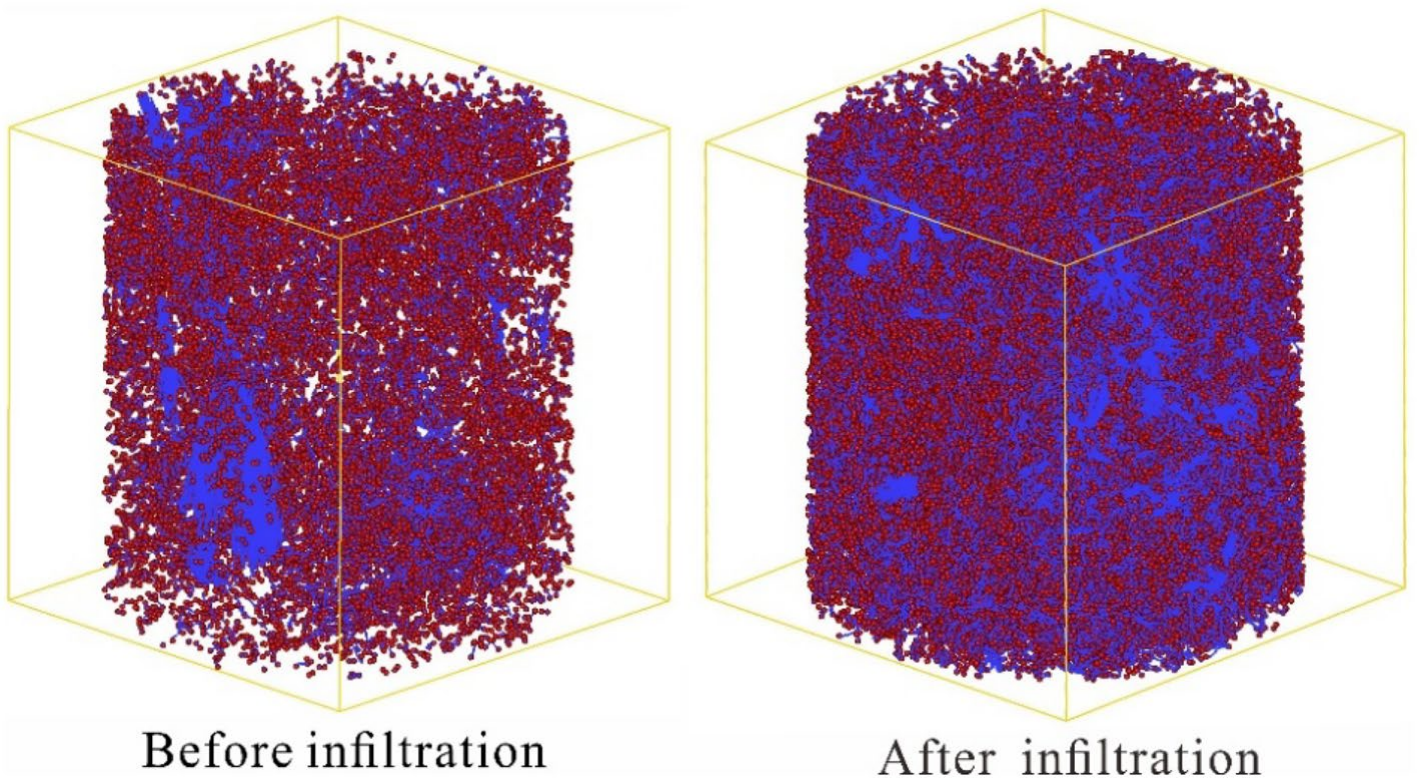
The pore network model before and after infiltration.

**Figure 13:**
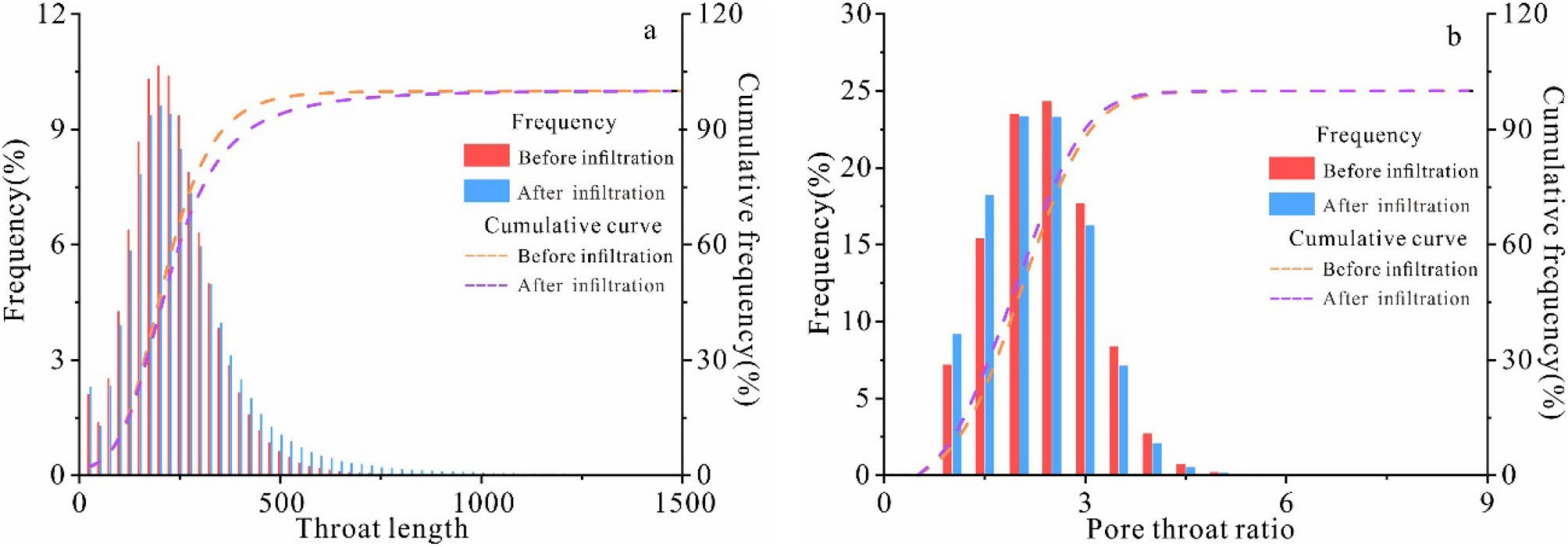
The distribution frequency of the throat length (**a**) and pore-throat ratio (**b**).

The pore network model of the undisturbed loess during infiltration was established using Avizo software. The model divided the different pore structures into pore and throat structures. The throat structures are channels in the soil that allow for easy water diffusion flow (Fig. 12). The pore-throat distribution of the undisturbed loess was calculated using the pore-throat ratio and the coordination number index in order to analyze the pore-throat structure change characteristics. The pore-throat ratio is the ratio of pores to throats, and the coordination number reflects the spatial center geometric characteristics of the pore structure. The higher the pore-throat ratio and coordination number, the more complex the pore structure and the less the water flows^44^ (Fig. 13). The pore-throat ratio and the coordination number of the undisturbed loess decrease after the infiltration tests, indicating that a specific scale of large-scale channels with good fluidity within the undisturbed loess is formed by the infiltrated water; therefore, the scale of the complex pore structure decreases within the undisturbed loess^44^.

The increase of a throat length than 400 μm is more apparent, reflecting the vertical development of the large pores in the undisturbed loess (Fig. 13a). The proportion of undisturbed loess with a smaller pore throat ratio than 1.5 after permeability test is higher, accounting for about 22.56% of all pore throats (Fig. 13b), indicating that the undisturbed loess has formed a more interconnected dendritic pore structure internally, with increased connectivity and volume scale between pores, developing into the main channels for water infiltration. The arrangement of soil particles in close contact with each other is easily changed by hydraulic action, thereby causing the soil structure to weaken to some extent. Changes in pore characteristics show that the undisturbed loess is sensitive to water infiltration due to an increase in the number of pores and the pore connectivity of the undisturbed loess, leading to a more dominant infiltration. Under the effect of water infiltration, the pore connectivity of undisturbed loess increases, which is more conducive to water entry and leads to soil structural damage. This is also why the undisturbed loess slope easily forms a slope slip along the dominant seepage channel, forming a collapsed settlement during long-term water infiltration.

## Discussions

Thick loess deposits can be considered "Kast soils" due to their susceptibility to internal erosion, including transport erosion and dissolution, which can cause catastrophic phenomena under the effects of water infiltration, resulting in nutrient loss and settlement in farmlands^2,3,5,6,9,33^. However, there is current controversy over how water enters loess, mainly focusing on dominant and piston inflow infiltration. Additionally, there are debates surrounding the consequences of fine particle migration, the structural changes caused by water entering loess, and the factors that affect these changes^9,33^.

### Water infiltration process

Water is a key factor that induces landslides in the loess area due to the loess's water sensitivity; however, the complexity of the loess structure and its properties result in the diversity and uncertainty of the water infiltration within the loess^9,45–47^. Many scholars have researched water infiltration problems within loess^26,46,48–50^. Some researchers believe that rainfall recharges the groundwater through dominant infiltration channels within the loess, such as vertical joints, tensile cracks, and sinkholes^26,46,51–53^. However, other scholars have pointed out that 2 m below the unsaturated loess, water can infiltrate down via unsaturated seepage or by virtue of water vapor. This effect was shown through field monitoring of rainfall infiltration in the loess^48^. Numerous research results found two types of infiltration in the loess^26,28,33,49^. One type is the unsaturated seepage from the surface to the underground^50,51^. Although the short-term infiltration depth is limited, the long-term unsaturated infiltration can recharge the groundwater. The other type is the rapid infiltration process via the dominant infiltration channels, controlled by the loess's joints and fissures. The surface water can quickly infiltrate deep into the loess stratum and recharge the groundwater^5,9^.

**Figure 14:**
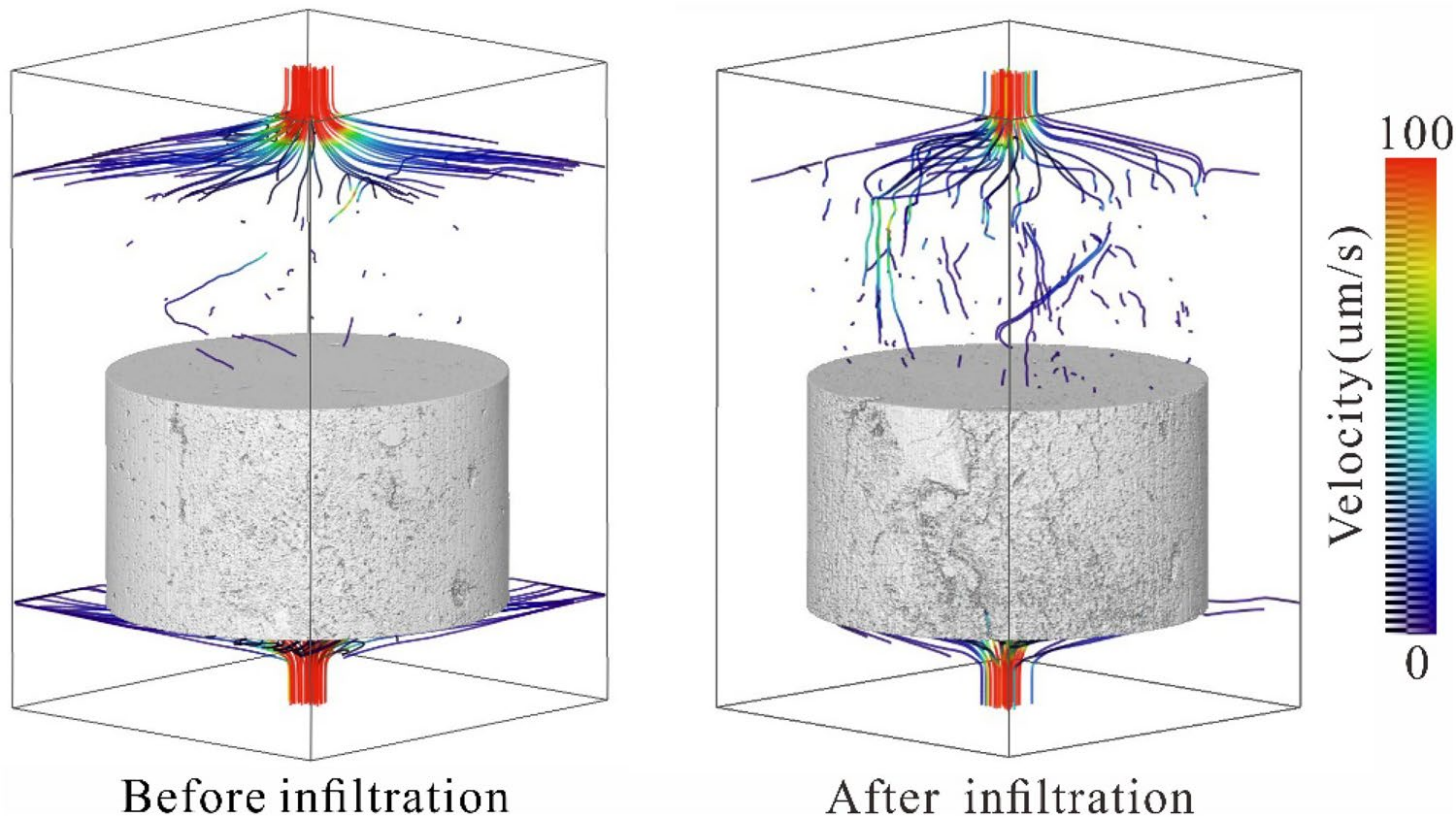
The distribution of the water flow velocity in the infiltration channel.

**Figure 15:**
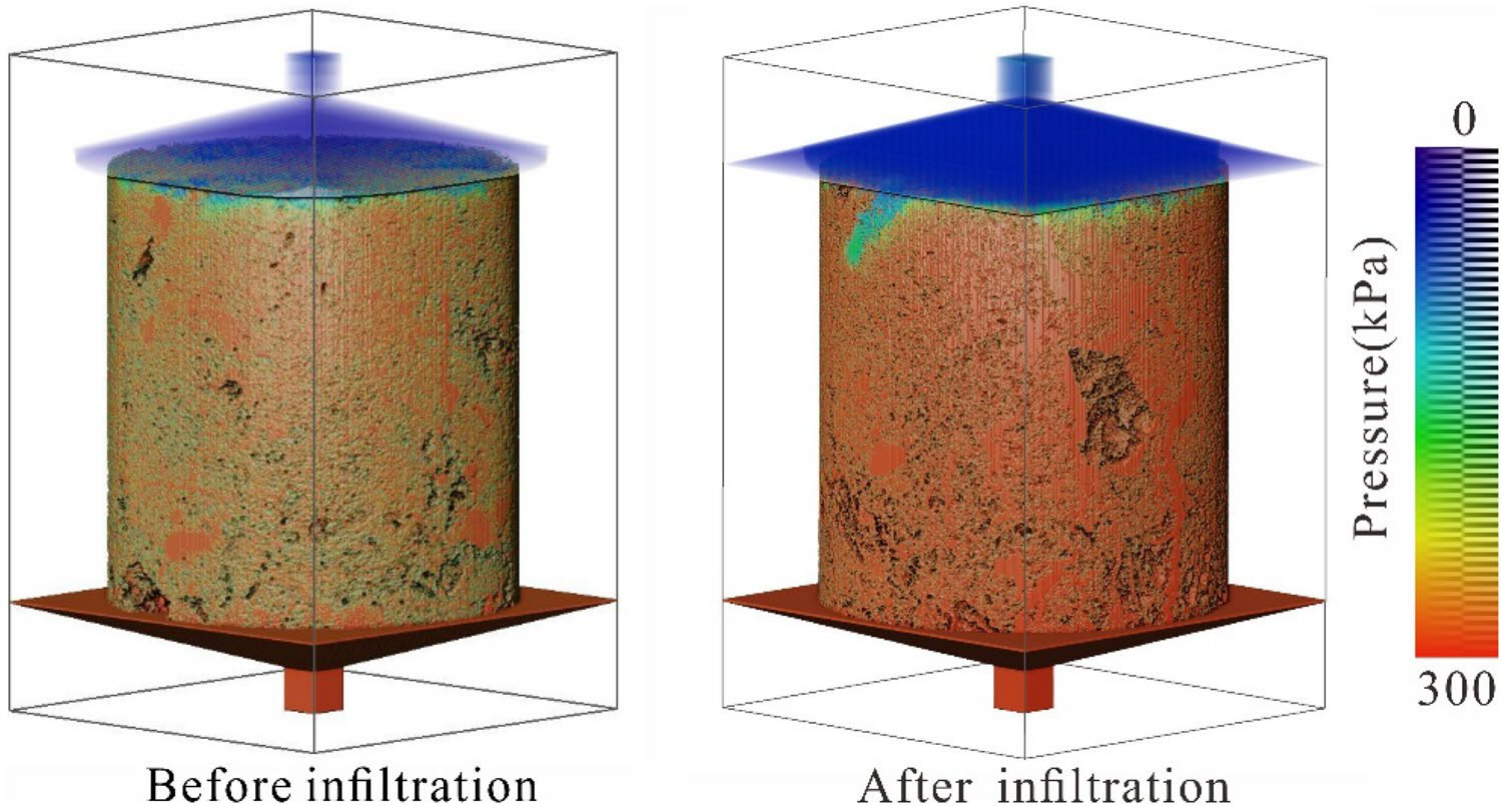
The distribution of the fluid pressure in the infiltration channel.

Through a large number of field investigations and indoor tests, it was found that the water enters the loess along the cracks, removing fine particles, destroying the structure of the loess, and forming a subterranean cave within the loess due to internal erosion. Using the Avizo software absolute seepage process simulation module, the seepage characteristics of the fracture and pipe flow are obtained via simulations and calculations according to the simplified Navier–Stokes equation^52^. Combined with the laboratory infiltration test, it was found that there are dominant channels in the undisturbed loess. The dominant channels continue to increase during water infiltration, resulting in a higher water flow velocity in the near-vertical channels (Fig. 14). From the simulation results of the fluid pressure changes (Fig. 15), it was found that due to continuous water infiltration, the fluid pressure of the pore channel in the undisturbed loess increases continuously. The increase of fluid pressure of the pore channel leads to the destruction of the loess structure and a decrease in loess strength. This process reflects the change of the loess structure under the action of water resulting in a decrease in strength; thus, inducing loess failure^35,53^.

### Collapsibility due to internal erosion

Loess is naturally deposited via the accumulation of wind-blown fine sand and clay components, and compared with other soil masses, loess has unique karst-like characteristics due to the aeolian transportation process during its formation. The karst-like nature of loess is characterized by the development of joints and fissures at a macroscopic level and unconsolidated and large cohesive strength when it is dry. However, when the loess is wet, the cohesive strength reduces significantly at the mesoscopic level, and aggregate particles and overhead pores are developed at the microscopic level^53^. Once the water infiltrates into the loess, the loess will undergo a significant reduction in strength and subsidence deformation^54^. This typical loess water-sensitive catastrophe phenomenon is sudden, discontinuous, and irreversible. This is the early failure stage of the loess slope and is triggered by water^13^. Many studies have been conducted on the apparent behavior of water-sensitive loess, such as collapsibility, softening, and liquefaction triggered by water. Various theories have been proposed, such as capillary force reduction, water film wedging, shear strength reduction, pulsation liquefaction, microstructural imbalance, salt solution, colloid insufficiency, uncompression, and structure failure^55,56^. Most of these theories explain the mechanism of loess water-sensitive catastrophe from a single point of view, such as physics or chemistry^9,38^. However, the process of dissolution, invasion, and transportation by water in the loess that affects the loess structure and induces the deformation and failure of the loess is still unclear. The structural changes caused by the internal erosion of loess are the key factors for collapsible subsidence, with the structural changes being primarily due to particle migration and chemical erosion^56,57^.

During the infiltration process of this experiment, with an increase in infiltration time, the fine particles were eroded by the water seeps from the undisturbed loess samples. Table 2 shows the X-ray diffraction pattern results of the fine particles eroded from the undisturbed loess. The Illite, Chlorite, Plagioclase, Potash feldspar, Dolomite, and Amphibole contents of the soil particles eroded from the undisturbed loess are minimal. In contrast, there is an increase in the quartz and calcite contents compared to the original loess sample.

**Table 2:** The X-ray diffraction patterns result from the fine particles eroded from the loess sample (%).

Sample	Illite	Chlorite	Quartz	Potassium feldspar	Plagioclase	Calcite	Amphibole	Kaolinite	Dolomite
Undisturbed loess	20.2	3.7	32.1	11.1	23.4	7.4	0.9	0.6	0.6
Fine particles were eroded by the water	8.3	2	56.5	5.7	13.7	11.8	0.8	0.6	0.5

The soil particles eroded from the undisturbed loess is 0.43 g and consist of clay, silt, and sand at a concentration of 21.91%, 73.565%, and 4.485%, respectively. From the fine particles that seep out from the soil sample, it can be seen that the migration of fine particles caused by water infiltration is not often considered clay particles but is mainly composed of fine particles with very low clay particle concentrations.

**Figure 16:**
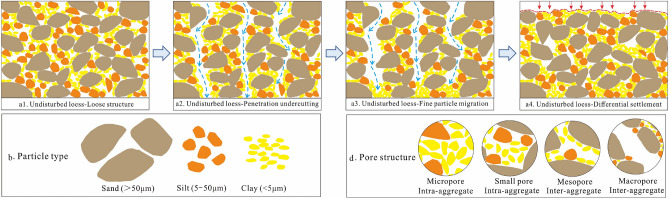
The collapsibility and subsidence due to loess structure destruction triggered by water infiltration.

Internal erosion is a phenomenon in which the fine particles gradually migrate through the pores between the coarse particles resulting in a damaged soil skeleton^58^. Moreover, the smaller fine particles inside the soil samples migrate preferentially^11^. The fine particles are then carried a certain distance by the water and are deposited, causing some pores to become blocked^15^. Coarse-grained particles play a role in the soil's skeletal structure. Fine particles are often filled between the coarse particles, such as clay minerals and soluble salts that are filled between the coarse and the fine particles, resulting in cementation^59^. During the infiltration test, the soluble salts in the soil are dissolved and leached, causing a disintegration of the fine particles in the cement. The disintegrated fine particles migrate downward through the pores between the coarse particles under the driving force of water infiltration; therefore, the fine particle contents increase in the downward position of the loess sample. The migration of the fine particles is an important cause of loess collapsibility and subsidence due to the destruction of the loess structure and an increase in pore water caused by the blocked pores (Fig. 16).

### Effects of loess salinity on structural changes

The typical characteristic of loess is that it contains numerous soluble salts compared to other soils^9^. The salts that migrate and are dissolved in water during water infiltration significantly destroy the loess structure. Many researchers have pointed out that the study of loess landslides from the aspects of soil physics is insufficient, neglecting the weakening of the mechanical parameters of the loess caused by the hydrochemistry, often leading to the overestimation of the slope's stability resulting in accidents from field investigations^60^. The effect of hydrochemistry on loess failure is still unclear; however, what is known is that the salinity of the loess in different regions varies greatly. The test results revealed that the loess of Qingyang mainly consisted of Na^+^, ${\text{SO}}_{{4}}^{{{\text{2{-}}}}}$ and ${\text{HCO}}_{{3}}^{ - }$, while the loess of Heifangtai mainly consisted of Na^+^, Ca^2+^, Mg^2+^, ${\text{CI}}^{ - }$ and ${\text{SO}}_{{4}}^{2-}$ and ${\text{HCO}}_{{3}}^{ - }$. It can be seen that the salt content of the loess in Heifangtai contains high concentrations of Na^+^ salt (Table 3).

**Table 3:** Ion concentration of the supernatant (mg/L).

Sample	${\text{Na}}^{+}$	${\text{K}}^{+}$	${\text{Ca}}^{2+}$	${\text{Mg}}^{2+}$	CI^−^	${\text{SO}}_{{4}}^{{{2} - }}$	${\text{HCO}}_{{3}}^{-}$	${\text{CO}}_{{3}}^{{{2} - }}$	${\text{NO}}_{{3}}^{ - }$
Qingyang	189.63	5.62	0.28	4.71	11.23	34.00	40.91	0.00	6.15
Heifangtai	613.34	16.20	149.06	40.28	232.85	522.00	19.61	0.00	33.94

In order to discuss the impact of salt content in loess on the structural changes caused by water infiltration, we conducted a water infiltration test using the same method on the Qingyang and Heifangtai loess, which have significant differences in salt content. The water ions that seeped out of the loess sample were tested to analyze the impact of salt dissolution caused by water infiltration on the loess structure. The settlement of the loess from the Heifangtai tableland was 3 cm (sample height 12 cm) after the infiltration test, while the loess sample from the Qingyang tableland did not undergo settlement deformation during the infiltration test. The loess with a high salt content also had significant structural strength^15,53,54^. The loess's structure and strength are significantly reduced with low salt content, and the residual strength becomes smaller after the infiltration test^38,61^. Therefore, the soil particles can overcome the resistance of the surrounding soil and cause collapsing deformation^34,59,61^. The permeated water's ion content was tested by collecting water during the infiltration process. The ion concentrations of the Heifangtai and Qingyang loess are shown in Table 4.

**Table 4:** Ion content carried by water infiltration (mmol).

Sample	Na^+^	K^+^	Ca^2+^	Mg^2+^	CI^−^	${\text{SO}}_{{4}}^{{{2} - }}$	${\text{HCO}}_{{3}}^{-}$	${\text{CO}}_{{3}}^{{{2} - }}$	${\text{NO}}_{{3}}^{ - }$
Qingyan	1.18	0.03	1.27	0.63	0.15	0.22	4.71	0.00	0.06
Heifangtai	22.49	0.42	5.857	1.79	16.17	17.61	2.04	0.15	0.01

As seen in Table 3, the salt content of Na^+^ Ca^+^, ${\text{Cl}}^{ - }$, and Ca^2+^ ions dissolved from the Heifangtai loess are much higher than the Qingyang loess. At the same time, the salt content of the Heifangtai tableland is higher than that of the Qingyang tableland. Therefore, during the water infiltration process, a large amount of salt from the Heifangtai loess was removed by dissolution during the infiltration test resulting in the thickness of the salt acting as a cement at the contact point of the particles, therefore, becoming smaller. Furthermore, the residual cementation strength cannot resist the external force that makes the particles move; therefore, the contact point cemented by the salt breaks, and the loess sample collapses and deforms. In contrast, the loess from the Qingyang tableland has less salt, and minimal structural strength was lost during the infiltration test. Its residual cementation strength is large, and it cannot overcome the resistance of the surrounding soil, thus, causing a deformation collapse. Moreover, the loess sample from the Qingyang tableland did not settle during the infiltration test.

Salt is loess's primary mineral, which is easily solubolized during water infiltration^9,10,38^. Therefore, the catastrophic mechanism of loess during water infiltration is more apparent than that of other rocks and soils. The reduction of strength and structural damage of loess caused by the chemical dissolution of the loess with high salt content is often much higher than that of the loess with low salinity and is the chemical reason for the loess's strength when dried; however, its strength significantly decreases after water infiltration^9,38^.

### Strength decreases due to water infiltration

**Figure 17:**
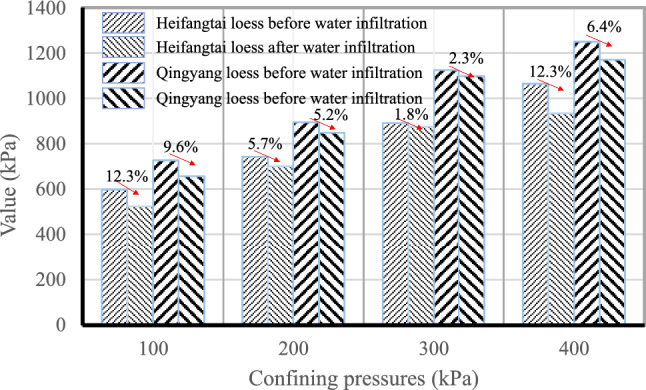
Unconfined compressive strength of Qingyang and Heifangtai loess before and after infiltration.

Numerous studies have analyzed the loess strength decrease due to water infiltration and focused on the weakness of the cementation effect due to the insoluble salts, soluble salts, and clay particles in the loess skeleton dissolution and migration, resulting in a gradually decreasing strength^7,9,38,62^. While the water infiltrates into the loess, it not only dissolves the soluble salts in the loess but also physically erodes the loess removing the fine particles and changing its structural characteristics. In order to analyze the impact of water infiltration on the strength of loess and compare the differences in strength attenuation caused by different chemical dissolutions, the same method was used to test the strength of the Heifangtai and Qingyang loess before and after infiltration under the same soil moisture content. Figure 17 shows the strength changes before and after infiltration using unconfined compressive strength method, defined as the strength of the specific against axial deformation without lateral stress. It can be seen that the strength of loess after infiltration decreases, but the magnitude of the decrease varies under different stress states. At the same time, under the conditions of 100, 200, and 400 kPa of Heifangtai loess, the strength offset amplitude of loess after infiltration is significantly greater than that of Qingyang loess, indicating that different salt content does affect the softening effect of water on the loess. For loess with significant salt content, the dissolution effect of water after entering will be greater, significantly changing the loess's structural state, thus reducing the loess strength. Therefore, the impact of water entering loess can be summarized as follows: infiltration—fine particle migration—chemical composition dissolution—structural damage—strength reduction, resulting in loess catastrophes.

## Conclusions

In this paper, the structural characteristics of the undisturbed loess before and after water infiltration are studied using CT, and water infiltration tests. The effect is studied using numerical simulation and mechanical tests. The process of water infiltration into the loess, the loess collapsibility mechanisms, and the salinity's influence on the loess structure are discussed. The results obtained are as follows:

1. The distribution frequency curve of the equivalent diameter of the particles moves to the right side of the coordinate axis. The migration of fine particles caused by water infiltration is not often considered clay particles but is mainly composed of fine particles.
2. The porosity of loess after infiltration increased by approximately 20% on average. The increase in the pore area ratio of the meso and the macropores is higher than that of the micro and small pores leading to more dominant infiltration.
3. The migration of fine particles is an essential cause of loess collapsibility and subsidence due to the destruction of the loess structure. The reduction of the strength and structural damage of loess caused by chemical dissolution with high salt is often much greater than that of loess with low salinity.

## Data Availability

The data supporting this study's findings are available from the corresponding author, Jianqi Zhuang, upon reasonable request.
